# Dataset describing the amino acid catabolism of *Thermoanaerobacter pseudethanolicus*

**DOI:** 10.1016/j.dib.2023.110017

**Published:** 2023-12-27

**Authors:** Johann Orlygsson, Sean Michael Scully

**Affiliations:** University of Akureyri, Faculty of Natural Resource Sciences, Borgir 2 v/ Norðurslóð, 600 Akureyri, Iceland

**Keywords:** Thermophilic, Branched-chain amino acids, Alcohols, and alcohols, Thiosulfate, Anaerobic

## Abstract

The dataset depicts the catabolism of branched-chain amino acids by *Thermoanaerobacter pseudethanolicus* in the presence of thiosulfate under different culture conditions. The results reveal that the strain can degrade all three branched-chain amino acids resulting in the production of their corresponding branched-chain fatty acids and branched-chain alcohols with the fatty acids always being the dominant product. The highest amounts of 2-methyl-1-butanol from isoleucine were at pH 6.5, liquid-gas ratio of 0.98, and at 20 mM thiosulfate concentration. A kinetic experiment of the branched-chain amino acids was done in the presence of thiosulfate as are data on selected enzyme activities related to alcohols and aldehydes. Finally, an NMR study using ^13^C1 methyl-1-butyrate during the degradation of leucine in the presence of thiosulfate was done to prove that the ^13^C1-methyl-1-butanol was indeed from its corresponding fatty acid.

Specifications Table

**Table:** 

Subject	Biology
Specific subject area	Microbiology
Data format	Raw
Type of data	Table, figure
Data collection	The anaerobic bacteria investigated were cultivated under various environmental conditions and both substrate and end-product formation were analysed using GC-FID, Perkin Elmer Clarus 580, GC-TCD, Perkin Elmer Autosystem XL, UV-Visible Spectroscopy, Bioscreen C (GrowthCurves Ltd, Finland) and Shimadzu UV-1800 UV-Visible Spectrometer, Bruker AV400 NMR Spectrometer.
Data source location	Institution: University of Akureyri Region: Akureyri, Iceland
Data accessibility	Repository name: Mendeley Data identification number: 10.17632/kpbmvxh88m.1 Direct URL to data: https://data.mendeley.com/datasets/kpbmvxh88m/1
Related research article	S.M. Scully, J. Orlygsson, Branched-chain amino acid catabolism of Thermoanaerobacter pseudoethanolicus reveals potential route to branched-chain alcohol formation, Extremophiles. 24 (2020) 121–133.

## 1 Value of the Data

• The data presents fermentation products from the catabolism of the branched-chain amino acids, namely leucine, isoleucine, and valine, using thiosulfate as an electron scavenging system by *Thermoanaerobacter pseudethanolicus*.
• The data set shows the influence of culture parameters (pH, partial pressure of hydrogen, initial thiosulfate concentration) on the fermentation of a branched-chain amino acid, L-isoleucine, in batch culture, as well as a kinetic experiment showing the formation of branched-chain fatty acids and alcohols from branched-chain amino acids.
• The enzyme activity data using alcohols and aldehydes as substrates suggest that the enzymes involved in the production of alcohols as end products from amino acid catabolism have a degree of promiscuity.
• The ^13^C NMR experiments reveal that a potential route for the production of the corresponding alcohols results from the reduction of the fatty acid to the alcohol under fermentative conditions.
• The fermentation end product data provides insight into the catabolism of amino acids in thermophilic environments such as hot springs where sulfur species are often present.
• Suggests that *Thermoanaerobacter* species are important parts of the nitrogen cycle within hot springs and other thermal environments and the use of alternative electron sinks such as fatty acids
• The data is useful for comparison of amino acid catabolism of other mesophilic and thermophilic anaerobes producing higher-value linear and branched-chain aliphatic alcohols.

## 2 Data Description

Similar to a number of mesophilic Clostridia, *Thermoanaerobacter pseudethanolicus* (DSM 2355) degrades the branched-chain amino acids (BCAAs) to a mixture of the corresponding branched-chain fatty acids (BCFA; major end product) and alcohols (BCOH; minor end product) in the presence of thiosulfate [1]. The influence of various culture parameters known to result in shifts in end product formation (such as liquid-gas phase ratio, pH, and the concentration of thiosulfate) was investigated in batch culture using isoleucine as a model BCAA.

The dataset contains eight tables (Tables final.docx), seven of which detail fermentation data, namely metabolic end products such as alcohols, fatty acids, and hydrogen, while the remaining table contains enzyme activities towards selected alcohol and aldehyde substrates. Each line details the experimental conditions for a given experiment with a data point for the analyte concentration in mmol per L presented as the average ± standard deviation measured at the indicated time. Additionally, one table summarizes the enzymatic activity of crude cell lysates towards alcohol and aldehyde substrates using NAD^+^ or NADP^+^ as a cofactor. Table 1 summarizes the production of fermentation products (BCFAs and BCOHs) from BCAAs (20 mM) with and without the addition of thiosulfate (40 mM). Similarly, Table 2 shows the effect of the initial pH of the fermentation medium on the catabolism of isoleucine while Table 3 details end-product formation when the strain was cultivated at various liquid-gas phase ratios, a proxy for gauging the influence of the partial pressure of hydrogen, ranging from a low L-G ratio with a large headspace to a high L-G ratio with limited headspace. Table 4 shows the impact of initial thiosulfate concentrations between 0 and 60 mM on the fermentation products from isoleucine catabolism. Additionally, detailed kinetic experiments were performed over a period of 7 days using all three of the BCAAs (valine, isoleucine, and leucine) as single substrates with thiosulfate addition as summarized in Table 5, Table 6, Table 7. Finally, the volumetric activities oxidative enzyme reactions of *T. pseudethanolicus* cultivated on various substrates using NAD^+^ and NADP^+^ as a cofactor during growth of the strain on leucine with thiosulfate, and leucine with and without thiosulfate but in the presence of 3-methyl-1-butyrate (20 mM) were performed (Table 8).

**Table 1:** Degradation of BCAA by *Thermoanaerobacter pseudethanolicus* with (40 mM) and without thiosulfate after 7 days of cultivation. Values represent the average of triplicate measures ± standard deviation.

Substrate	**Analyte** (mmol/L)								BCAA degraded (%)	Carbon balance (%)	OD	pH
	Ethanol	BCOH	Acetate	BCFA	H_2_	H_2_S	S_2_O_3_	BCAA				
Control	0.79 ± 0.08	< 0.1	4.05 ± 0.16	< 0.1	3.58± 0.46	ND	ND	ND	ND	ND	0.19± 0.06	4.3± 0.2
Control + S_2_O_3_	0.04± 0.00	< 0.1	3.14± 0.21	< 0.1	0.12± 0.04	0.40± 0.14	0.00	ND	ND	ND	0.31± 0.05	4.9± 0.1
Valine	0.83± 0.06	< 0.1	2.31± 0.05	0.77± 0.04	4.23± 0.32	ND	ND	19.20± 0.21	4.0	xx	0.21± 0.04	5.2± 0.0
Valine + S_2_O_3_	0.26± 0.05	1.07± 0.03	8.17± 0.30	11.23± 1.11	0.20± 0.12	8.89± 1.10	0.00	7.70± 0.07	65.0	100.0	1.09± 0.15	5.8± 0.1
Leucine	0.73± 0.08	< 0.1	2.45± 0.18	0.56± 0.06	4.11± 0.22	ND	ND	19.40± 0.14	3.0	xx	0.27± 0.09	6.6± 0.2
Leucine + S_2_O_3_	0.31± 0.04	1.21± 0.18	7.35± 0.12	10.30± 0.21	0.30± 0.14	11.06± 0.82	0.00	8.48± 0.38	57.6	110.0	1.14± 0.17	6.8± 0.0
Isoleucine	0.67± 0.06	< 0.1	2.61± 0.18	0.34± 0.04	3.99± 0.25	ND	ND	19.50± 0.23	2.5	xx	0.34± 0.02	7.3± 0.1
Isoleucine + S_2_O_3_	0.41± 0.13	0.72± 0.34	7.40± 0.12	9.77± 0.83	0.33± 0.10	11.11± 0.34	0.00	9.50± 0.27	52.5	100.0	1.16± 0.13	7.6± 0.1

**Table 2:** Influence of pH on the fermentation of isoleucine (20 mM) and thiosulfate (20 mM) by *Thermoanaerobacter pseudethanolicus* after 14 days of cultivation. Values represent the average of triplicate measures± standard deviation.

Initial pH	**Analyte** (mmol/L)								Ile degraded (%)	Carbon balance (%)	OD	pH
	Ethanol	2-methyl-1-butanol	Acetate	2-methyl-1-butyrate	H_2_	H_2_S	S_2_O_3_	Ile				
4.0	0.00± 0.00	0.00± 0.00	1.21± 0.24	0.00± 0.00	0.07± 0.01	4.36± 0.48	16.51	17.31± 0.38	13.5	86.6	0.03± 0.00	4.3± 0.2
4.5	0.00± 0.00	0.00± 0.00	2.37± 0.13	0.00± 0.00	0.05± 0.01	6.31± 0.51	14.38	18.31± 0.41	8.5	91.6	0.04± 0.01	4.9± 0.1
5.0	0.00± 0.00	0.00± 0.00	2.60± 0.40	2.02± 0.51	0.05± 0.02	9.87± 1.07	15.43	13.20± 0.21	34.0	76.1	0.18± 0.01	5.2± 0.0
5.5	0.26± 0.05	3.45± 0.47	4.38± 0.28	15.81± 1.37	0.35± 0.08	10.41± 1.22	<0.50	0.34± 0.07	98.3	98.0	0.21± 0.03	5.8± 0.1
6.0	0.63± 0.08	4.21± 0.19	8.21± 0.22	16.25± 0.34	0.41± 0.09	11.58± 0.72	<0.50	0.64± 0.14	96.8	105.5	0.22± 0.02	6.6± 0.2
6.5	0.51± 0.11	4.36± 0.56	7.64± 0.31	15.34± 0.21	0.32± 0.04	12.49± 0.68	<0.50	1.39± 0.38	93.1	105.5	0.22± 0.03	6.8± 0.0
7.0	0.67± 0.06	3.94± 0.39	7.91± 0.49	16.71± 0.67	0.34± 0.07	12.08± 2.41	<0.50	1.01± 0.23	95.0	108.3	0.23± 0.01	7.3± 0.1
7.5	0.41± 0.13	4.18± 0.28	8.43± 0.27	16.57± 0.83	0.51± 0.12	10.59± 1.24	<0.50	1.27± 0.27	93.7	110.1	0.24± 0.02	7.6± 0.1
8.0	0.33± 0.03	4.09± 0.40	8.13± 0.13	16.38± 0.49	0.39± 0.05	11.89± 0.33	<0.50	4.22± 0.41	78.9	123.5	0.22± 0.01	8.2± 0.1
8.5	0.10± 0.01	4.10± 0.27	8.25± 0.21	15.14± 0.57	0.27± 0.07	11.30± 0.19	<0.50	3.61± 0.62	82.0	114.3	0.18± 0.02	8.8± 0.0
9.0	0.24± 0.13	1.84± 0.14	7.84± 0.39	11.12± 0.74	0.05± 0.02	13.21± 0.36	<0.50	8.13± 1.07	59.4	105.5	0.16± 0.02	9.3± 0.1

**Table 3:** Influence of liquid-gas phase ratio concentration on the fermentation of isoleucine (20 mM) and thiosulfate (20 mM|) by *Thermoanaerobacter pseudethanolicus* after 14 days of cultivation. Values represent the average of triplicate measures  ±  standard deviation.

L-G	**Analyte** (mmol/L)								Ile degraded (%)	Carbon balance (%)	OD	pH
	Ethanol	2-methyl-1-butanol	Acetate	2-methyl-1-butyrate	H_2_	H_2_S	S_2_O_3_	Ile				
0.05	2.14± 0.37	1.82± 0.63	6.24± 0.31	17.34± 0.34	0.27± 0.05	8.51± 0.51	<0.50	0.56± 0.17	97.22	98.6	0.24± 0.01	7.3± 0.2
0.34	1.97± 0.27	1.91± 0.22	5.76± 0.66	18.11± 0.67	0.57± 0.11	9.35± 0.23	<0.50	0.64± 0.27	96.80	103.3	0.23± 0.02	7.1± 0.1
0.98	2.14± 0.34	3.37± 0.38	5.81± 0.41	17.58± 0.45	1.23± 0.23	11.87± 1.21	<0.50	0.79± 0.14	96.05	108.7	0.27± 0.04	7.4± 0.1
2.08	2.43± 0.39	3.01± 0.54	4.98± 0.37	16.34± 0.32	1.49± 0.17	10.67± 0.87	<0.50	0.23± 0.05	98.85	97.9	0.27± 0.03	7.2± 0.1
5.4	2.28± 0.13	3.12± 0.34	3.94± 0.28	16.19± 0.17	1.57± 0.37	12.34± 1.94	<0.50	2.15± 0.43	89.25	107.3	0.25± 0.04	7.3± 0.0

**Table 4:** Influence of thiosulfate concentration on the fermentation of isoleucine (20 mM) by *Thermoanaerobacter pseudethanolicus* after 14 days of cultivation. Values represent the average of triplicate measures± standard deviation.

[S_2_O_3_] (mM)	**Analyte** (mmol/L)								Ile degraded (%)	Carbon balance (%)	OD	pH
	Ethanol	2-methyl-1-butanol	Acetate	2-methyl-1-butyrate	H_2_	H_2_S	S_2_O_3_	Ile				
0	0.34± 0.02	0.00± 0.00	2.83± 0.17	1.23± 0.06	0.40± 0.00	4.89± 0.51	<0.50	18.63± 2.74	6.85	99.3	0.20± 0.01	7.2± 0.1
10	2.21± 0.34	1.43± 0.34	4.91± 0.27	12.31± 0.07	1.52± 0.35	6.24± 1.32	<0.50	10.41± 0.68	47.95	120.8	0.24± 0.04	7.3± 0.1
20	3.57± 0.20	2.51± 0.62	6.83± 0.30	15.71± 0.43	1.14± 0.24	13.78± 3.47	<0.50	0.16± 0.04	99.20	91.9	0.23± 0.02	7.1± 0.0
30	3.14± 0.35	2.17± 0.24	7.39± 0.47	16.21± 0.21	0.67± 0.31	18.52± 2.81	<0.50	0.34± 0.07	98.30	93.6	0.34± 0.04	7.4± 0.2
40	2.07± 0.42	1.83± 0.31	7.81± 0.37	17.51± 0.49	0.86± 0.24	21.47± 4.46	<0.50	0.17± 0.08	99.15	97.6	0.29± 0.03	7.3± 0.1
50	1.42± 0.20	1.63± 0.38	8.44± 0.21	17.63± 0.72	0.21± 0.14	25.46± 5.24	<0.50	0.24± 0.05	98.80	97.5	0.34± 0.04	7.6± 0.3
60	1.81± 0.31	1.11± 0.27	8.63± 0.10	18.49± 1.01	0.19± 0.04	32.85± 4.30	<0.50	0.13± 0.11	99.35	98.7	0.32± 0.04	7.5± 0.2

**Table 5:** Fermentation kinetics of valine (20 mM) in the presence of thiosulfate (20 mM) by *Thermoanaerobacter pseudethanolicus*. Values represent the average of triplicate measures± standard deviation.

Time (h)	**Analyte** (mmol/L)							% AA degraded	Carbon balance (%)	OD
	Ethanol	2-methyl-1-propanol	Acetate	2-methyl-1-propionate	H_2_	S_2_O_3_	Val			
4	0.86± 0.12	0.00± 0.00	2.26± 0.32	0.00± 0.00	0.32± 0.02	20.00± 0.00	20.00± 0.00	0.0	100.0	0.21± 0.02
8	0.60± 0.14	0.00± 0.00	3.05± 0.12	0.00± 0.00	0.45± 0.13	15.37± 0.98	20.00± 0.00	0.0	100.0	0.50± 0.04
12	1.23± 0.12	0.00± 0.00	3.03± 0.14	0.36± 0.05	1.07± 0.28	10.78± 0.53	20.00± 0.00	0.0	101.8	0.43± 0.05
18	0.85± 0.06	0.32± 0.02	3.71± 0.11	1.48± 0.09	1.14± 0.22	6.96± 0.36	20.00± 0.00	0.0	109.0	0.27± 0.04
24	0.20± 0.04	0.47± 0.05	3.94± 0.26	2.48± 0.14	1.59± 0.40	3.31± 0.46	18.37± 0.39	8.2	106.6	0.27± 0.01
30	0.44± 0.14	0.65± 0.06	4.45± 0.28	3.65± 0.23	1.44± 0.18	1.39± 0.40	17.34± 0.24	13.3	108.2	0.33± 0.04
36	1.07± 0.05	0.71± 0.06	4.58± 0.09	4.40± 0.17	2.48± 0.37	0.77± 0.18	15.96± 1.09	20.2	105.4	0.40± 0.05
48	0.39± 0.09	0.95± 0.04	5.11± 0.12	6.66± 0.37	2.23± 0.63	0.19± 0.07	13.89± 3.62	30.6	107.5	0.53± 0.08
60	0.52± 0.04	0.87± 0.08	5.08± 0.16	7.79± 0.84	1.94± 0.31	0.00± 0.00	13.14± 2.95	34.3	109.0	0.76± 0.10
72	0.80± 0.22	0.89± 0.14	5.58± 0.27	10.61± 0.77	2.31± 0.49	0.00± 0.00	10.42± 0.94	47.9	109.6	0.35± 0.04
120	0.11± 0.05	0.62± 0.10	5.83± 0.07	16.35± 0.38	1.83± 0.42	0.00± 0.00	3.02± 0.63	84.9	100.0	0.33± 0.01
168	0.00± 0.00	2.12± 0.17	6.21± 0.14	17.03± 0.41	1.07± 0.37	0.00± 0.00	0.34± 0.07	98.3	97.5	0.32± 0.02

**Table 6:** Fermentation kinetics of isoleucine (20 mM) in the presence of thiosulfate (20 mM) by *Thermoanaerobacter pseudethanolicus*. Values represent the average of triplicate measures± standard deviation.

Time (h)	**Analyte** (mmol/L)							% AA degraded	Carbon balance (%)	OD
	Ethanol	2-methyl-1-butanol	Acetate	2-methyl-1-butyrate	H_2_	S_2_O_3_	Ile			
4	1.95±0.42	0.00± 0.00	1.53± 0.45	0.48± 0.12	0.17± 0.05	20.00± 0.00	20.00± 0.00	0.0	102.4	0.31± 0.05
8	4.62± 0.40	0.00± 0.00	2.87± 0.06	0.29± 0.03	0.63± 0.19	20.00± 0.00	20.00± 0.00	0.0	101.5	0.45± 0.06
12	5.87± 0.44	0.00± 0.00	3.05± 0.09	0.46± 0.10	1.43± 0.21	13.99± 0.29	20.00± 0.00	0.0	102.3	0.45± 0.08
18	6.73± 0.36	0.00± 0.00	3.65± 0.11	2.31± 0.36	1.72± 0.43	10.04± 0.54	17.37± 0.39	0.0	98.4	0.39± 0.07
24	6.10± 0.26	0.87± 0.04	3.97± 0.23	4.31± 0.20	1.63± 0.38	6.40± 0.37	16.38± 0.76	18.1	107.8	0.36 ± 0.01
30	5.56± 0.16	1.32± 0.07	4.53± 0.11	7.04± 0.43	1.84± 0.55	2.79± 0.33	12.79± 0.96	36.1	103.5	0.53± 0.07
36	5.92± 0.03	1.60± 0.04	4.55± 0.15	8.74± 0.29	2.07± 0.20	0.40± 0.14	11.55± 0.30	42.3	109.5	0.64± 0.09
48	4.54± 0.57	2.30± 0.27	4.99± 0.30	14.00± 1.14	2.43± 0.42	0.16± 0.05	8.01 ± 0.88	60.0	121.6	0.67± 0.10
60	3.70± 0.33	2.44± 0.16	5.09± 0.10	16.48± 0.74	1.20± 0.38	0.00± 0.00	7.44± 0.34	62.8	131.8	0.63± 0.03
72	3.04± 0.43	2.11± 0.32	5.46± 0.37	20.83± 1.25	1.40± 0.23	0.00± 0.00	5.39± 0.27	73.1	119.4	0.61± 0.16
120	1.39± 0.44	2.39± 0.29	5.98± 0.16	25.76± 0.94	1.03± 0.13	0.00± 0.00	4.05± 0.07	79.8	116.1	0.40± 0.05
168	1.10± 0.20	4.07± 0.24	6.08± 0.17	24.34± 1.67	0.79± 0.05	0.00± 0.00	1.27± 0.13	93.7	118.4	0.34± 0.07

**Table 7:** Fermentation kinetics of leucine (20 mM) in the presence of thiosulfate (20 mM) by *Thermoanaerobacter pseudethanolicus*. Values represent the average of triplicate measures± standard deviation.

Time (h)	**Analyte** (mmol/L)							% AA degraded	Carbon balance (%)	OD
	Ethanol	3-methyl-1-butanol	Acetate	3-methyl-1-butyrate	H_2_	S_2_O_3_	Leu			
4	5.38± 0.93	0.00± 0.00	1.25± 0.16	0.63± 0.15	0.34± 0.12	20.00± 0.00	20.00± 0.00	0.0	103.1	0.42± 0.07
8	6.56± 0.44	0.00± 0.00	2.57± 0.64	0.65± 0.12	0.78± 0.33	20.00± 0.00	20.00± 0.00	0.0	103.3	0.65± 0.06
12	7.89± 0.31	0.00± 0.00	2.59± 0.21	0.54± 0.05	1.07± 0.24	13.34± 1.13	20.00± 0.00	0.0	102.7	0.66± 0.05
18	8.00± 0.10	0.00± 0.00	3.21± 0.25	1.66± 0.40	1.40± 0.47	11.06± 0.19	20.00± 0.00	0.0	108.3	0.34± 0.09
24	7.52± 0.10	0.57± 0.01	3.51± 0.53	2.94± 0.79	1.66± 0.23	11.65± 3.61	20.00± 0.00	0.0	114.7	0.28± 0.02
30	6.87± 0.19	0.70± 0.07	3.59± 0.17	4.68± 0.93	1.83± 0.56	5.58± 1.77	20.00± 0.00	0.0	113.4	0.31± 0.03
36	6.68± 0.14	0.85± 0.07	3.88± 0.15	6.20± 0.86	2.25± 0.38	0.53± 0.19	20.00± 0.00	0.0	108.6	0.33± 0.11
48	5.41± 0.45	1.21± 0.10	4.10± 0.28	9.58± 0.98	2.41± 0.47	0.00± 0.00	20.00± 0.00	0.0	110.2	0.46± 0.06
60	5.60± 0.24	1.85± 0.80	4.50± 0.66	12.61± 3.44	2.87± 0.69	0.00± 0.00	12.68± 0.97	36.6	132.5	0.60± 0.13
72	4.80± 0.04	1.59± 0.13	4.80± 0.26	16.01± 1.90	1.63± 0.43	0.00± 0.00	9.07± 1.50	54.7	133.4	0.47± 0.11
120	3.04± 0.42	1.07± 0.47	5.40± 0.33	22.67± 1.61	1.28± 0.18	0.00± 0.00	7.79± 0.47	61.1	127.6	0.39± 0.06
168	2.06± 0.37	1.43± 0.34	5.71± 0.27	23.37± 1.32	1.32± 0.21	0.00± 0.00	3.22± 0.24	83.9	120.1	0.41±0.02

**Table 8:** Enzyme activities (alcohol and aldehyde dehydrogenase activities) using either NAD^+^ or NADP^+^ as a factor of culture of *T. pseudethanolicus* grown on leucine (20 mM) supplemented thiosulfate (20 mM), leucine (20 mM) supplemented 3-methyl-1-butyrate (20 mM), and leucine (20 mM) supplemented 3-methyl-1-butyrate (20 mM) and thiosulfate (20 mM) after 20 h at 65 °C. Enzyme activities performed by adding 50 µL of cell lysate at a microplate containing 135 µL of reaction solution (containing 330 µM of NAD^+^ or NADP^+^, 330 µM of NBT, 0.13% w/v gelatin, and 5.5 mM of substrate in 50 mM of Tris-HCl buffer, pH 8.0) followed by 15 µL of 80 µM. Change in absorbance (580 nm) monitored and used to calculate activity where 1 unit of activity corresponds to 1 µmol/min of turn over. Values represent the average of triplicate determinations± standard deviation.

Growth conditions	Substrate	Cofactor			
		NAD^+^		NADP^+^	
		Specific activity (mU/mg protein)	Relative activity (%)	Specific activity (mU/mg protein)	Relative activity (%)
Leucine + S_2_O_3_	Control	2.05± 0.24	NA	1.93± 0.11	NA
	EtOH	40.25± 6.38	100.0^a^	0.00± 0.00	NA
	1-PrOH	18.29± 4.48	45.4^a^	0.00± 0.00	NA
	2-PrOH	40.64± 17.63	101.0^a^	25.01± 9.67	NA
	1-BuOH	13.23± 11.91	32.9^a^	27.09± 3.86	NA
	2-BuOH	75.40± 14.71	187.3^a^	31.40± 6.41	NA
	2-Me-1-PrOH	31.96± 7.57	79.4^a^	37.17± 3.15	NA
	1-Pentanol	70.75± 8.69	175.8^a^	26.17± 9.57	NA
	2-Pentanol	23.27± 2.46	57.8^a^	44.98± 13.51	NA
	2-Me-1-BuOH	14.59± 4.91	36.2^a^	26.25± 7.84	NA
	3-Me-1-BuOH	36.30± 6.14	90.2^a^	45.15± 2.86	NA
	1-Hexanol	24.00± 1.75	59.6^a^	0.00± 0.00	NA
	2-Hexanol	27.33± 5.06	67.9^a^	0.00± 0.00	NA
	1-Heptanol	45.85± 4.38	113.9	0.00± 0.00	NA
	1-Octanol	38.33± 8.21	95.2	0.00± 0.00	NA
	Acetaldehyde	16.43± 0.00	40.8^b^	0.00± 0.00	NA
	Propionaldehyde	84.06± 6.23	208.8^b^	0.00± 0.00	NA
	Butyraldehyde	48.75± 8.97	121.1^b^	0.00± 0.00	NA
	2-Methyl-1-propionaldehyde	163.45± 16.48	406.1^b^	85.80± 13.87	NA
	Pentanaldehyde	124.01± 28.70	308.1^b^	41.31± 1.16	NA
	3-Methyl-butyraldehyde	128.06± 19.24	318.1^b^	76.25± 15.31	NA
	Hexaldehyde	80.59± 4.91	200.2^b^	94.72± 17.01	NA
Leucine + 3-Methyl-1-butyrate	Control	2.05± 0.24	NA	1.93± 0.11	NA
	EtOH	11.97± 2.92	100.0^a^	50.31± 7.37	100.0^a^
	1-PrOH	15.89± 3.81	132.7^a^	23.31± 5.25	46.3^a^
	2-PrOH	30.23± 3.48	252.5^a^	11.25± 2.43	22.4^a^
	1-BuOH	29.30± 6.94	244.7^a^	23.73± 12.39	47.2^a^
	2-BuOH	40.03± 14.60	334.3^a^	25.36± 3.43	50.4^a^
	2-Me-1-PrOH	33.41± 8.87	279.1^a^	0.00± 0.00	0.0^a^
	1-Pentanol	30.63± 10.27	255.8^a^	12.62± 3.71	25.1^a^
	2-Pentanol	24.53± 2.34	204.8^a^	19.52± 6.71	38.8^a^
	2-Me-1-BuOH	31.12± 6.44	259.9^a^	17.62± 3.32	35.0^a^
	3-Me-1-BuOH	29.28± 0.89	244.5^a^	16.72± 0.16	33.2^a^
	1-Hexanol	27.90± 2.50	233.0^a^	0.00± 0.00	0.0^a^
	2-Hexanol	18.69± 0.68	156.1^a^	0.00± 0.00	0.0^a^
	1-Heptanol	13.75± 1.73	114.8^a^	9.40± 5.63	18.7^a^
	1-Octanol	34.61± 9.22	289.1^a^	0.00± 0.00	0.0^a^
	Acetaldehyde	0.00± ± 0.00	NA	38.11± 6.39	100.0^b^
	Propionaldehyde	54.79± 8.16	NA	34.37± 3.68	90.2^b^
	Butyraldehyde	10.19± 2.22	NA	42.17± 2.17	110.7^b^
	2-Methyl-1-propionaldehyde	117.69± 48.42	NA	52.14± 5.22	136.8^b^
	Pentanaldehyde	2.76± 0.61	NA	33.14± 2.79	87.0^b^
	3-Methyl-butyraldehyde	0.00± 0.00	NA	18.16± 0.00	47.6^b^
	Hexaldehyde	0.00± 0.00	NA	0.00± 0.00	0.0^b^
Isoleucine + 3-methyl-1-butyrate + S_2_O_3_	Control	2.05± 0.24	NA	1.93± 0.11	NA
	EtOH	47.99± 6.29	100.0^a^	25.59± 13.36	100.0^a^
	1-PrOH	20.02± 8.81	41.7^a^	27.71± 2.31	108.3^a^
	2-PrOH	15.06± 4.31	31.4^a^	45.23± 8.87	176.8^a^
	1-BuOH	43.64± 4.50	90.9^a^	33.29± 21.40	130.1^a^
	2-BuOH	30.10± 1.13	62.7^a^	35.29± 18.58	137.9^a^
	2-Me-1-PrOH	50.01± 15.19	104.2^a^	39.66± 12.39	155.0^a^
	1-Pentanol	35.14± 18.46	73.2^a^	27.71± 7.30	108.3^a^
	2-Pentanol	26.12± 2.25	54.4^a^	61.16± 2.76	239.0^a^
	2-Me-1-BuOH	9.81± 1.31	20.4^a^	30.90± 18.37	120.7^a^
	3-Me-1-BuOH	11.25± 13.26	23.5^a^	52.66± 13.73	205.8^a^
	1-Hexanol	12.31± 2.74	25.7^a^	34.80± 4.71	136.0^a^
	2-Hexanol	21.34± 4.50	44.5^a^	55.52± 4.78	217.0^a^
	1-Heptanol	33.49± 2.35	69.8^a^	18.69± 10.60	73.0^a^
	1-Octanol	37.38± 5.42	77.9^a^	25.59± 12.06	100.0^a^
	Acetaldehyde	27.18± 0.92	100.0^b^	0.00± 0.00	NA
	Propionaldehyde	8.13± 6.66	29.9^b^	0.00± 0.00	NA
	Butyraldehyde	3.39± 0.65	12.5^b^	37.92± 1.98	NA
	2-Methyl-1-propionaldehyde	7.40± 0.92	27.2^b^	36.02± 3.09	NA
	Pentanaldehyde	40.50± 3.47	149.0^b^	133.67± 26.94	NA
	3-Methyl-butyraldehyde	14.44± 4.01	53.1^b^	38.50± 8.60	NA
	Hexaldehyde	34.25± 4.74	126.0^b^	50.37± 9.48	NA

a Relative to ethanol.

b Relative to acetaldehyde, NA – not applicable.

**Fig. 1:**
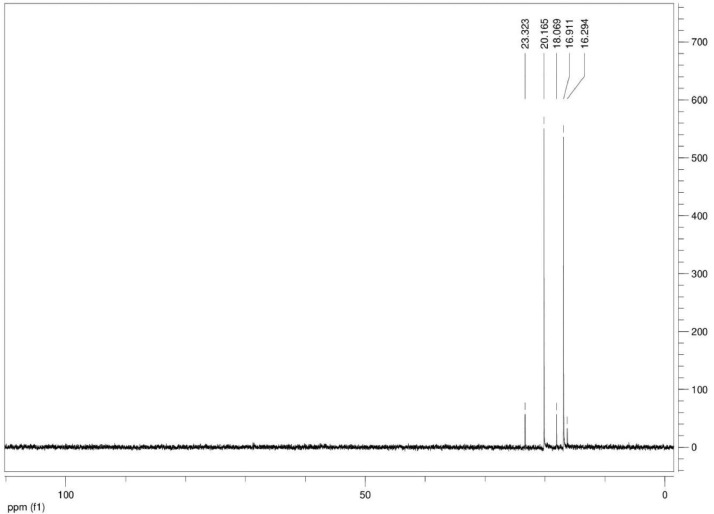
Spectrogram of ^13^C NMR spectra from *T. pseudethanolicus* (DSM 2355) culture broth containing 20 mM of ^13^C1 glucose after 168 h of fermentation.

**Fig. 2:**
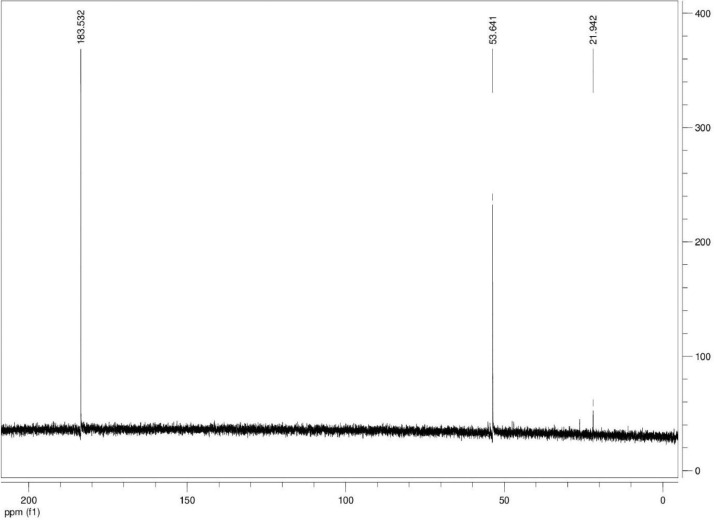
Spectrogram of ^13^C NMR spectra from *T. pseudethanolicus* (DSM 2355) culture broth containing 20 mM of ^13^C2 leucine + thiosulfate (40 mM) after 14 d of fermentation.

**Fig. 3:**
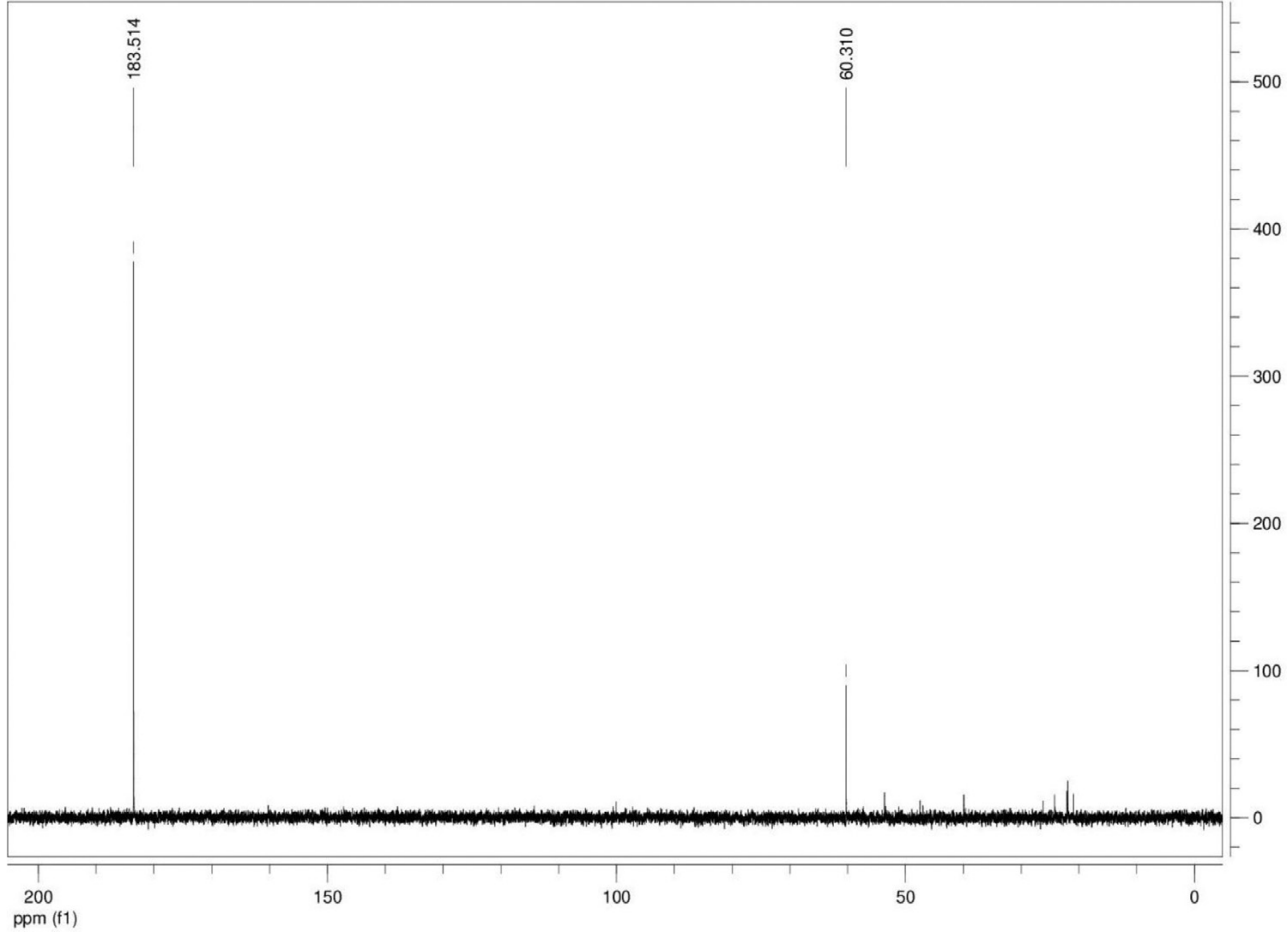
Spectrogram of ^13^C NMR spectra from *T. pseudethanolicus* (DSM 2355) culture broth containing 20 mM of ^13^C1 3-methyl-1-butyrate + leucine (20 mM) after 14 d of fermentation.

**Fig. 4:**
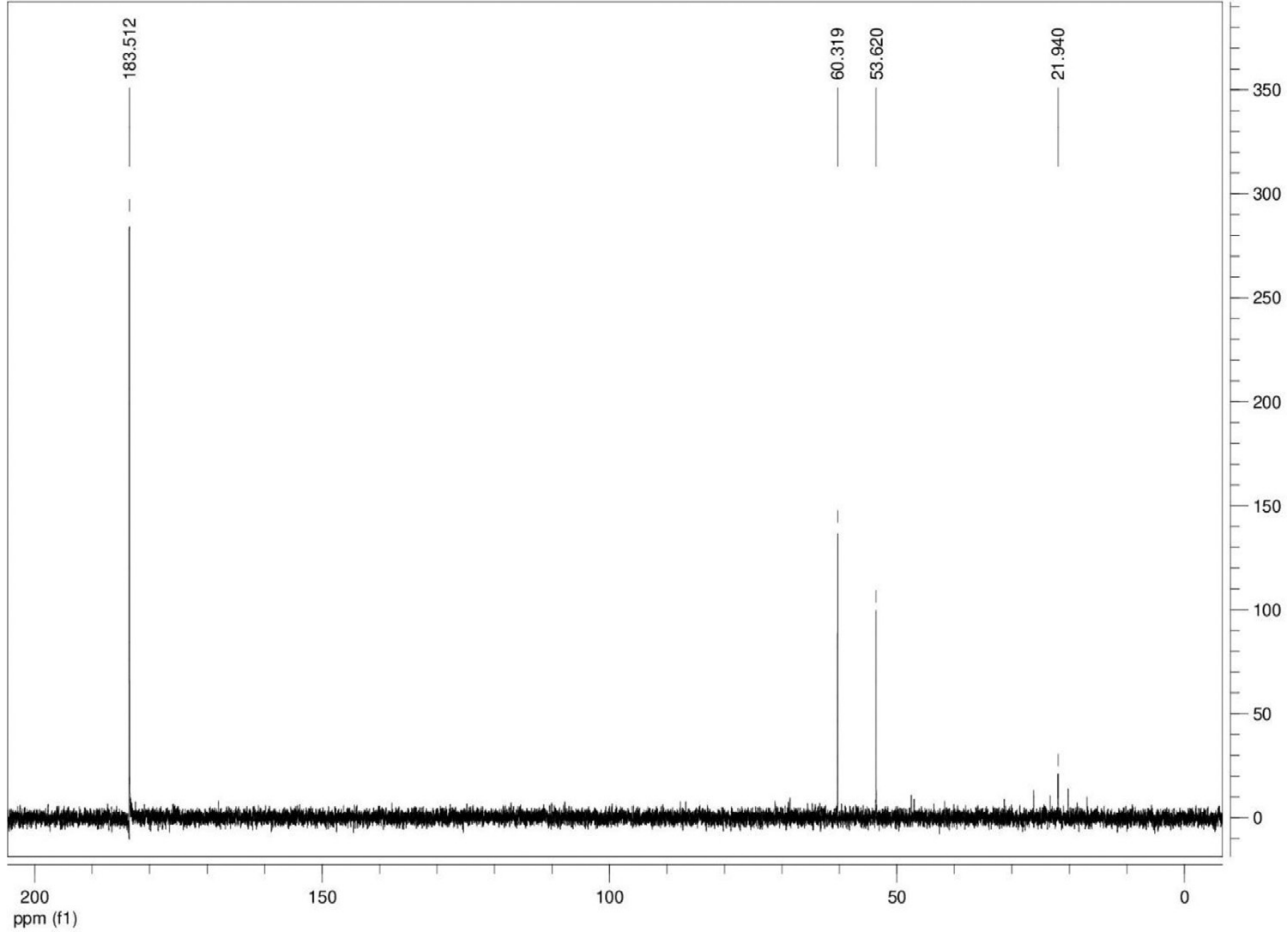
Spectrogram of ^13^C NMR spectra from *T. pseudethanolicus* (DSM 2355) culture broth containing 20 mM of ^13^C1 3-methyl-1-butyrate + S_2_O_3_ (40 mM) after 14 d of fermentation.

**Fig. 5:**
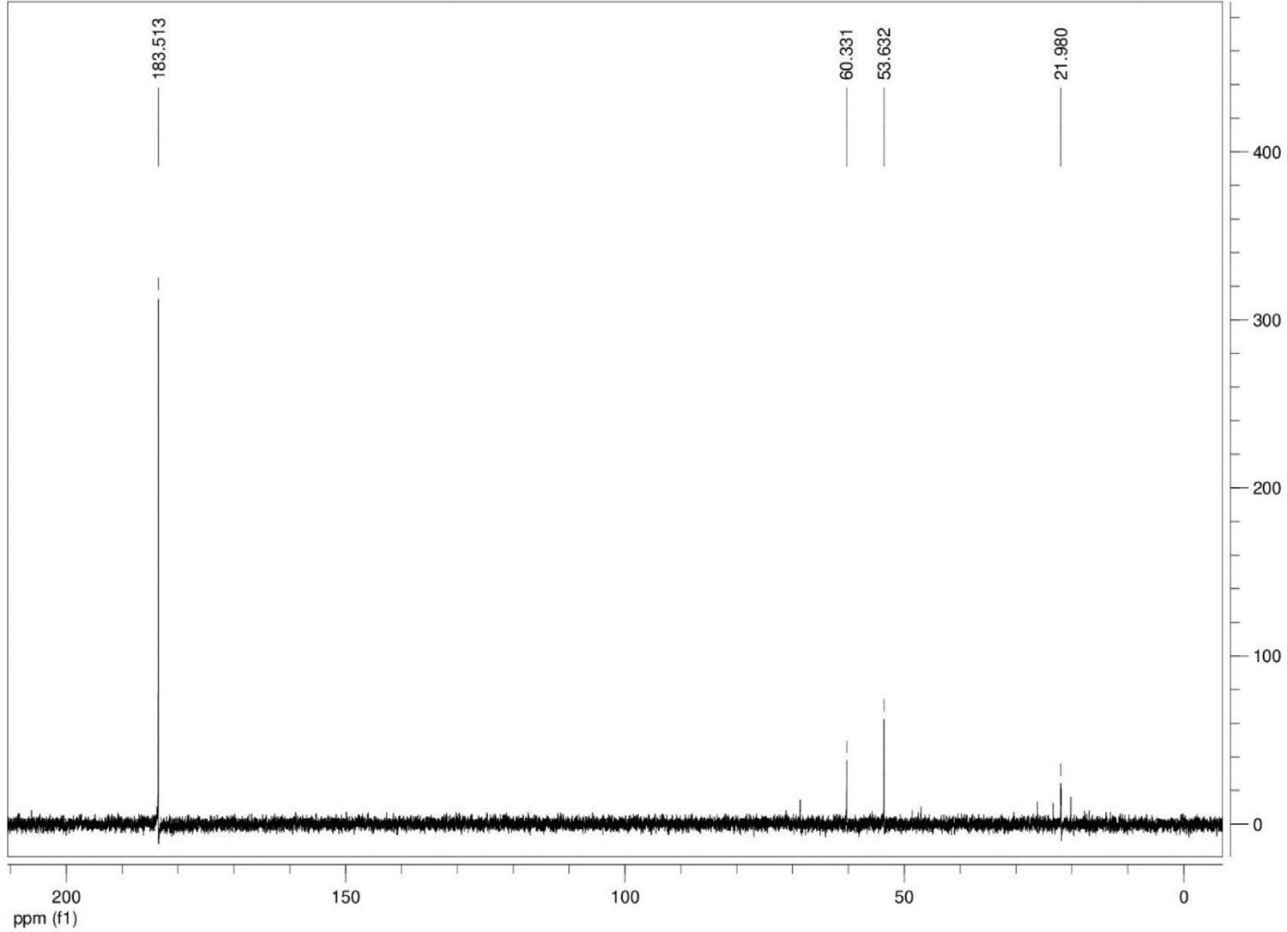
Spectrogram of ^13^C NMR spectra from *T. pseudethanolicus* (DSM 2355) culture broth containing 20 mM of ^13^C1 3-methyl-1-butyrate after 14 d of fermentation.

**Fig. 6:**
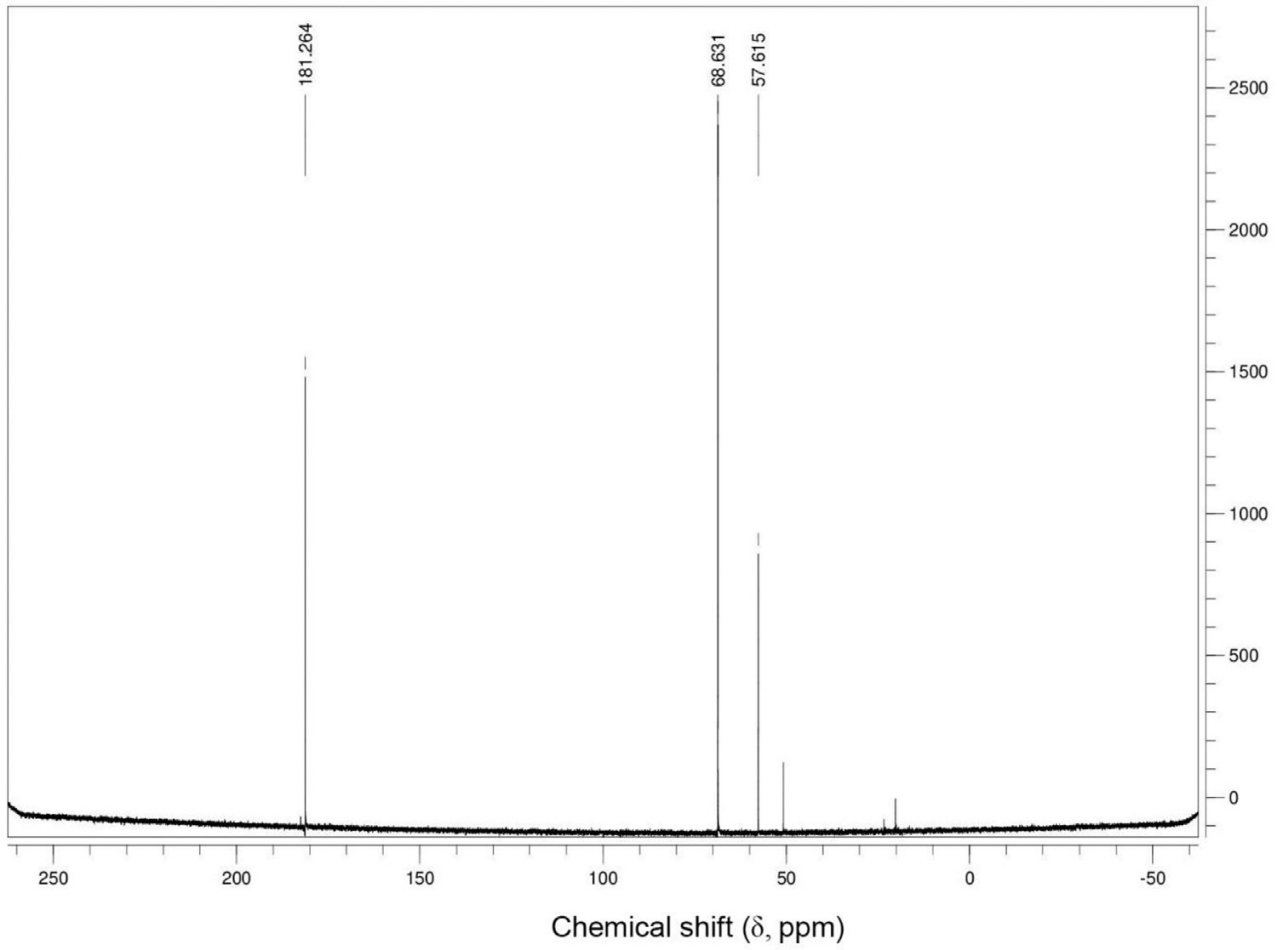
Spectrogram of ^13^C NMR spectra from *T. pseudethanolicus* (DSM 2355) culture broth containing 20 mM of ^13^C1 3-methyl-1-butyrate and glucose (20 mM) after 14 d of fermentation.

Additionally, ^13^C NMR spectrograms (Figures final.docx) demonstrate the conversation of selected isotopically labeled substrates. The ^13^C NMR spectra (Fig. 1, Fig. 2, Fig. 3, Fig. 4, Fig. 5, Fig. 6) highlight end products from fermentation experiments using selected ^13^C-labeled substrates; ^13^C1 glucose (Fig. 1) a combination of ^13^C2-labeled leucine which yields a combination of ^13^C1-labeled 3-methyl-1-butyrate (183.5 ppm, dominant end product) and 3-methyl-1-butanol (53.6 ppm, minor end product) using sodium thiosulfate as an electron scavenger (Fig. 2). Fig. 3 demonstrates the use of ^13^C1-labeled 3-methyl-1-butyrate serving as a terminal electron acceptor in the presence of leucine as an electron donor which demonstrates that ^13^C1-labeled 3-methyl-1-butanol as an end product and electron sink. Fig. 4, Fig. 5, Fig. 6 show the ^13^C NMR spectra of other isotopically labeled control experiments.

## 3 Experimental Design, Materials and Methods

### 3.1 General methods

Yeast extract was obtained from Difco; nicotinamide cofactors were obtained from Megazyme while all other reagents were acquired from Sigma-Aldrich. Nitrogen gas was acquired from AGA and contained less than 5 ppm O_2_. Isotopically labelled substrates were obtained from Cambridge Isotope Laboratories (MA, USA).

### 3.2 Microorganism and cultivation

*Thermoanaerobacter pseudethanolicus* (DSM 2355) was obtained from DSMZ culture collection and freezer stocks maintained in Basal Mineral (BM) medium supplemented with 30% v/v glycerol and stored at −20 °C. The strain was routinely cultivated in serum bottles or Hungate tubes using the Basal Mineral (BM) medium prepared as previously described [2] using the Hungate technique [3,4]. The content of BM, media preparation and sterilization has been described earlier [2]. Vitamins, trace element solution and, substrates were added separately through sterilized filter (0.45 µm, polyethersulfone or cellulose acetate) after autoclaving. Substrates were provided at a final concentration of 20 mM unless explicitly stated otherwise. All cultivations were performed at 65 °C and at pH of 7.0 with a liquid–gas (L–G) ratio of 1:1 without agitation except when stated otherwise. Inoculation of the strain was taken from the exponential growth phase using an inoculation volume of 2% (v/v). All cultivations were performed as triplicates and fermentation products were quantified after five days of cultivation unless stated otherwise.

### 3.3 Fermentation of branched-chain amino acids in presence and absence of thiosulfate

The capability of *T. pseudethanolicus* to catabolize branched-chain amino acids, namely valine, leucine, and isoleucine, was evaluated at concentration of 20 mM with and without thiosulfate supplementation (40 mM). Cultivations were carried out serum bottles (117.5 mL nominal volume) for 7 days at which time fermentation broth (1 mL) and headspace gas (0.2 mL) was removed for analysis.

### 3.4 Effect of initial pH on isoleucine fermentation

*T. pseudethanolicus* was cultivated at various initial pH values (4.0 to 9.0 in 0.5 unit increments) using isoleucine (20 mM) as a model BCAA plus the addition of thiosulfate (20 mM) in 16 × 150 mm Hungate tubes for 14 days. Samples were taken for analysis as described in the previous section.

### 3.5 Effect of liquid-gas phase ratio

*T. pseudethanolicus* was grown in anaerobic bottles (118.5 mL nominal volume) which were filled with specific volumes of BM medium containing isoleucine (20 mM) and thiosulfate (20 mM); final media volume of 10.0, 30.0, 59.25, 80.0, and 100.0 mL of media to afford L-G ratios of 0.09, 0.34, 1.00 2.12, and 5.41, respectively. Fermentation products were analyzed as above after 14 days.

### 3.6 Effect of initial thiosulfate concentration

The impact of thiosulfate concentration on isoleucine (20 mM) fermentation was performed with the addition of between 0 to60 mM of thiosulfate in 10 mM increments. The experiments were performed Hungate tubes (16 × 150 mm). End products were quantified after 14 days as described above.

### 3.7 Kinetic experiments

Time-course studies of the degradation of leucine, isoleucine, and valine (20 mM) with thiosulfate (20 mM) were performed in 118.5 mL anaerobic bottles over 7 days. 1 mL aliquots of fermentation broth and headspace gas were periodically for analysis as described above.

### 3.8 Enzyme assays

Cells for enzymatic assays were grown in 1 L serum bottles fitted with butyl rubber septa containing 500 mL of BM media containing Leu (20 mM) and electron acceptor (20 mM, 3-methyl-1-butyrate and/or sodium thiosulfate). After 18 h of cultivation, 5 mg of potassium dithionate was added to protect against oxygen and the cells were harvested by centrifugation (4700 rpm, 0–4 °C, 15 min). Cell pellets were transferred to a serum bottle and stored under a nitrogen atmosphere at -80 °C prior to analysis. Cells were lysed and oxidative enzymes assays using C2–C8 alcohols and aldehydes with NAD^+^ and NADP^+^ using an Nitro blue tetrazolium -linked assays in microtiter plates described by [1,6].

Briefly, 50µL of diluted enzyme solution, 135 µL of reagent solution (330 µM NAD^+^ or NADP^+^, 330 µL NBT, 0.13% w/v gelatine, 5 mM CaCl_2_ in 50 mM Tris buffer, pH 8.0) containing 5.5 mM of the relevant alcohol, and 15 µL of 10X phenazine methosulfate solution (80 µM) were dispensed into microplates. Samples were incubated anaerobically at 65 °C in a Bioscreen C (GrowthCurves, Ltd, Finland) and read every 2 min at 580 nm. A standard curve was generated by the use of NADH and NADPH. ADH activity was calculated according to the formula below, where *v* is the sample volume in mL and *t* is the time in minutes.

$$ADH\;activity\;\left(\frac{mU}{mL}\right)=\frac{nmol\;NADH}{v\cdot t}=nmol\;NADHx2$$

### 3.9 Analytical methods

Hydrogen in the bottle headspace and carboxylic acids and alcohols were measured via gas chromatography as previously described [2]. Thiosulfate, hydrogen sulfide, protein, and amino acids were quantified colorimetrically as previously communicated [5]. Optical density (OD) was measured at 600 nm with a Shimadzu UV-1800 UV-Visible spectrophotometer with quartz cuvette (*l*=1 cm) against a water blank.

### 3.10 ^13^C-labled experiments

*T. pseudethanolicus* was cultivated on BM medium supplemented with 20 mM of ^13^C2 leucine with and without the addition of sodium thiosulfate and/or 3-methyl-1-butyrate (20 mM) for two weeks as previously described [1]. Also, the strain was cultivated with ^13^C1 glucose as a control for 48 h and similarly analyzed.

## Limitations

Not applicable.

## Ethics Statement

The authors have read and follow the ethical requirements for publication in Data in Brief and confirming that the current work does not involve human subjects, animal experiments, or any data collected from social media platforms.

## CRediT authorship contribution statement

**Johann Orlygsson:** Supervision, Conceptualization, Methodology, Writing – original draft. **Sean Michael Scully:** Methodology, Writing – original draft, Investigation, Software.

## Data Availability

Dataset describing the amino acid catabolism of Thermoanaerobacter pseudethanolicus (Original data) (Mendeley Data)
